# Development of Lipid-Shell and Polymer Core Nanoparticles with Water-Soluble Salidroside for Anti-Cancer Therapy

**DOI:** 10.3390/ijms15033373

**Published:** 2014-02-25

**Authors:** Dai-Long Fang, Yan Chen, Bei Xu, Ke Ren, Zhi-Yao He, Li-Li He, Yi Lei, Chun-Mei Fan, Xiang-Rong Song

**Affiliations:** 1 State Key Laboratory of Biotherapy, West China Hospital, Sichuan University, Chengdu 610041, Sichuan, China; E-Mails: fangdailongtwozero@126.com (D.-L.F.); yanzai1112@sina.com (Y.C.); xb1990625@126.com (B.X.); heyaode@163.com (Z.-Y.H.); 13981836037@139.com (Y.L.); fanchunmeiok@163.com (C.-M.F.); 2 Department of Pharmaceutical Sciences, University of Nebraska Medical Center, Omaha, NE 68198, USA; E-Mail: renkemallee@gmail.com; 3 College of Chemistry and Environment Protection Engineering, Southwest University for Nationalities, Chengdu 610041, Sichuan, China; E-Mail: lilihes@163.com

**Keywords:** salidroside, lipid-shell and polymer-core nanoparticles (LPNPs), PLGA, antitumor

## Abstract

Salidroside (Sal) is a potent antitumor drug with high water-solubility. The clinic application of Sal in cancer therapy has been significantly restricted by poor oral absorption and low tumor cell uptake. To solve this problem, lipid-shell and polymer-core nanoparticles (Sal-LPNPs) loaded with Sal were developed by a double emulsification method. The processing parameters including the polymer types, organic phase, PVA types and amount were systemically investigated. The obtained optimal Sal-LPNPs, composed of PLGA-PEG-PLGA triblock copolymers and lipids, had high entrapment efficiency (65%), submicron size (150 nm) and negatively charged surface (−23 mV). DSC analysis demonstrated the successful encapsulation of Sal into LPNPs. The core-shell structure of Sal-LPNPs was verified by TEM. Sal released slowly from the LPNPs without apparent burst release. MTT assay revealed that 4T1 and PANC-1 cancer cell lines were sensitive to Sal treatment. Sal-LPNPs had significantly higher antitumor activities than free Sal in 4T1 and PANC-1 cells. The data indicate that LPNPs are a promising Sal vehicle for anti-cancer therapy and worthy of further investigation.

## 1. Introduction

Salidroside (Sal), a main active component in the roots of Rhodiola species, exhibits a wide range of pharmacological properties including antitumor [1], neuroprotection [2,3], cardiovascular protection [4,5] and anti-oxidation [6,7]. Among these, the antitumor effect of Sal has been verified in many tumor cell lines such as human neuroblastoma cells [8], bladder carcinoma cells [9] and breast carcinoma cells [10]. However, despite the high *in vitro* anti-cancer potency, the *in vivo* antitumor efficacy of Sal has been significantly restricted by poor oral absorption and low tumor cell uptake. Sal has a high water-solubility. It is cleared quickly form the circulation, with a half-life of only ~0.5 h in rats after intravenous injection [11]. Additionally, the broad distribution of Sal by conventional formulation [12] might cause severe side effects due to the absence of tumor tissue selectivity [13,14].

In order to solve these issues, several Sal delivery systems using nanotechnology have been developed. Sal was encapsulated into pH sensitive mesoporous silica nanoparticles with poly (acrylic acid) shell-layers. This system could control the release of Sal. However, the entrapment efficiency (EE) was only 25.33% [15]. To enhance the EE, a liposome-Sal system was developed with 45% EE [16]. Hydrophilic Sal was incorporated into the internal aqueous core of the liposomes, but the instability of the lipid bilayer and short diffusion route made it easy to leak out. Our group constructed Sal-chitosan nanoparticles [17]. In spite of the increased EE (70.15%) and stability, the nonspecific electrostatic interaction between the positive charged nanoparticles and negatively charged normal cells membrane decreased the tumor targeting efficacy [18].

Lipid-shell and polymer core nanoparticles (LPNPs) combine the characteristics of polymer nanoparticles and liposomes. The dual advantages of the vesicular and particulate make it a promising drug carrier with high biocompatibility, stability and favorable pharmacokinetic profile [19]. Drugs could be efficiently encapsulated in the polymer core and/or between the lipid bilayers, and therefore increase the payload capability. Introduction of the polymer core might delay the diffusion of the drug and increase the stability of the lipid shell, thereby enhancing the drug EE and system stability. There are many studies developing LPNPs [20,21]. It is relatively easy to load hydrophobic drugs into LPNPs with high EE, because both the polymer core and the lipid bilayer could encapsulate drugs. However, it is challenging to achieve high EE and decent particle size when incorporating hydrophilic drugs into LPNPs. In order to solve the problems mentioned above, the main aim of this study was to develop Sal LPNPs with high drug entrapment efficiency, small particle size and increased tumor cell uptake. A modified emulsification-solvent-evaporation method was employed to prepare LPNPs. The parameters of formulation were systematically investigated. The optimal Sal loaded LPNPs (Sal-LPNPs) were comprehensively characterized. The *in vitro* antitumor activity of Sal-LPNPs was assessed in Sal sensitive cell lines.

## 2. Results and Discussion

### 2.1. Characterization of the Synthesized Polymer

**Figure 1.**
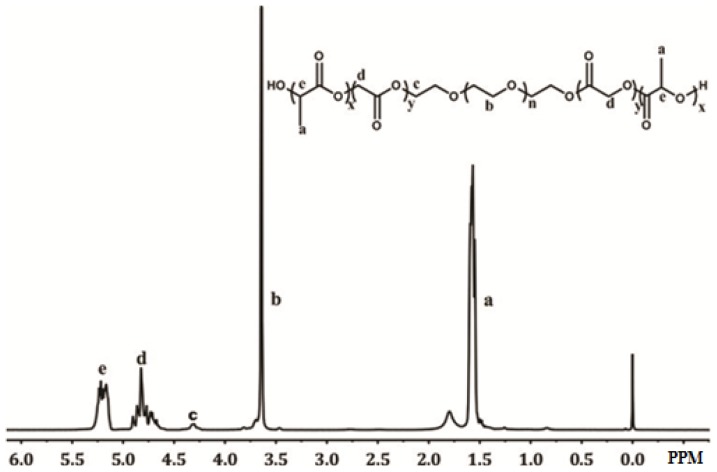
The ^1^H NMR spectrum of PLGA-PEG-PLGA copolymer. The proton peaks of a, b, c, d, e indicated δ 1.55 (–OCH(C*H_3_*)CO–), δ 3.64 (–OC*H_2_*CH_2_–), δ 4.30 (–OCH_2_*CH_2_*OCOCH_2_O–), δ 4.82 (–OC*H_2_*CO–) and δ 5.21 (–OC*H*(CH_3_)CO–), respectively. The *x*, *y* and *n* represented the repeating number of d,l-lactide, glycoside and PEG unit, respectively.

To systemically investigate the effect of different types of polymers on Sal encapsulation, poly(lactic acid-co-glycolic acid)-poly(ethylene glycol)-poly(lactic acid-co-glycolic acid) (PLGA-PEG-PLGA), one of the triblock copolymers which was used widely because of its biodegradability and good safety [22], cannot be obtained and was thus successfully synthesized by ROP with a high yield (80%) according to ^1^H NMR spectra. As shown in Figure 1, the proton peaks at 1.55 and 5.21 were ascribed to the methyl group (–OCH(C*H_3_*)CO–) and methine group (–OC*H*(CH3)CO–) of LA, respectively. The proton peak at 4.82 was assigned to the methylene group (–OC*H_2_*CO–) of GA, whereas the proton peaks at 3.64 and 4.30 were attributed to the methylene group (–OC*H_2_*CH_2_–) and (–OCH_2_C*H_2_*OCOCH_2_O–) of PEG. PLGA-PEG-PLGA with different molecular weights could be obtained by adjusting the reaction temperature and time according to the literature [23]. PLGA-PEG-PLGA with molecular weights of 7 and 15 KD were prepared at 150 °C for 6 h and 160 °C for 8 h, respectively. The molecular weight (*M*_W_) of PLGA-PEG-PLGA was estimated by ^1^H NMR. When the reaction time extended from 6 to 8 h and reaction temperature increased from 150 to 160 °C, it was obvious that the peak intensity ratio of the methene proton signal (5.21 ppm (–OC*H*(CH3)CO–) corresponding to the LA segments) or the methylene proton signal (4.82 ppm (–OC*H_2_*CO–) corresponding to the GA segments) to the methylene proton signal (3.64 ppm (–OC*H_2_*CH_2_–) corresponding to the PEG segments) increased proportionally.

### 2.2. Preparation of Sal-LPNPs

Sal-LPNPs were constructed successfully by a simple and controllable double emulsification method. According to our preliminary experiment, the highest EE could be achieved with lecithin/cholesterol at the weight ratio of 5/1 (data not shown) which additionally enabled Sal-LPNPs to be prepared easily. This optimal lecithin/cholesterol mass ratio was consistent with the literature reported by Fan *et al.* [24]. Accordingly, such a lipid combination was directly applied to optimize the processing parameters of Sal-LPNPs preparation. The power and time of sonication were also fixed at optimal values on the basis of our previous studies [25]. The other processing parameters were systemically investigated including the polymer types, organic phase, PVA types and content.

**Figure 2.**
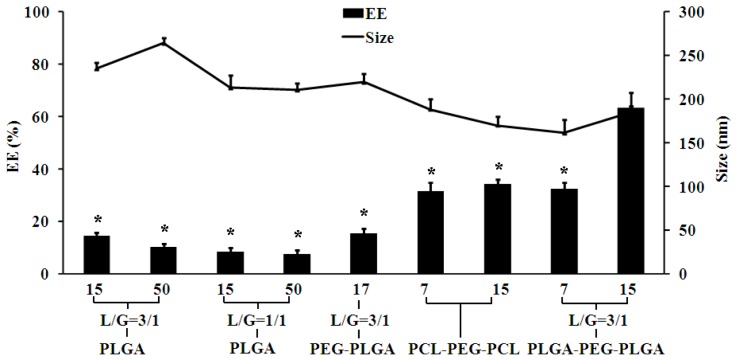
The effect of polymer types on the entrapment efficiency (EE) and size of Sal-LPNPs (mean ± SD, *n* = 3). * indicated there was significant difference (*p* < 0.05) compared to PLGA-PEG-PLGA (15 KD) group. The polymer type and *M*_W_ were shown on the *X* axis. L/G was the lactide/glycoside molar ratio in PLGA or polymers containing PLGA block.

**Figure 3.**
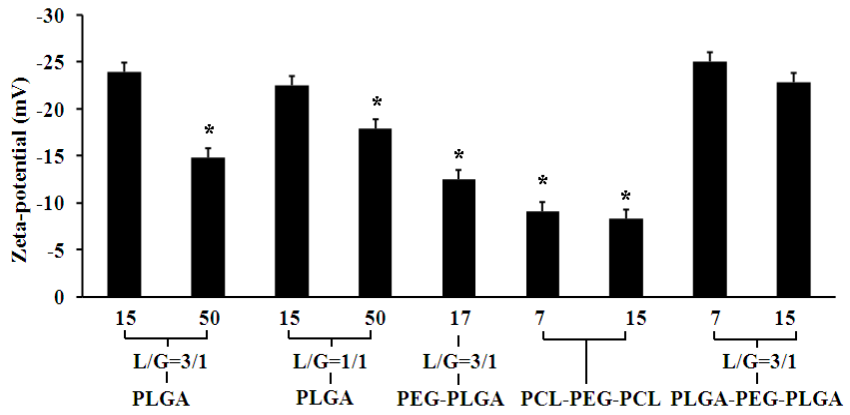
The effect of polymer types on the zeta-potential of Sal-LPNPs (mean ± SD, *n* = 3). * indicated there was significant difference (*p* < 0.05) compared to PLGA-PEG-PLGA (15 KD) group. The polymer type and *M*_W_ (KD) were shown on the *X* axis. L/G was the lactide/glycoside molar ratio in PLGA or polymers containing PLGA block.

Firstly, the effects of copolymer types on EE, particle size, particle distribution and zeta-potential were investigated. Four types of PLGA polymer with different glycolic acid/lactic acid molar ratio (L/G) and *M*_W_ were firstly used to prepare Sal-LPNPs. As shown in Figure 2, all the EEs of Sal using the four PLGA were less than 20%, among which PLGA (*M*_W_ = 15 KD, L/G = 3/1) achieved the highest EE. So PEG 2000 (hydrophilic block) modified PLGA (PEG-PLGA) was selected to produce Sal-LPNPs, and the EE increased slightly. To further improve the EE of Sal in Sal-LPNPs, four types of triblock copolymers with the same middle-block PEG 2000 were investigated. Water-soluble Sal might have a higher interaction with polymers with hydrophilic block. Therefore, PEGylated polymers with increased hydrophilicity could entrap Sal into LPNPs [26,27] more efficiently. Triblock copolymers including poly(e-caprolactone)-PEG-poly(e-caprolactone) (PCL-PEG-PCL) and PLGA-PEG-PLGA attained the higher EE, which might originate from the special spatial structure of polymers that formed in LPNPs basic skeleton. In the W/O coarse emulsion droplets, the intermediate PEG block might extend into the inner aqueous phase, while PLGA blocks in the two ends of the polymer molecules might reversely stretch into the outer organic phase and form a relatively firm network structure due to hydrophobic interaction. It would cause the formation of PEG protuberance towards the inner aqueous phase and thus promote the hydrophilic interaction of PEG with Sal (as shown in Figure 10C) [28]. PLGA-PEG-PLGA with the high *M*_W_ (15 KD) had larger particle size than the other triblock copolymer and hence higher EE. Larger nanoparticles containing high *M*_W_ PLGA-PEG-PLGA can increase the diffusion length of Sal, and thereby reduce the drug loss through diffusion and increasing EE [25]. This phenomenon can be explained by the decreased net shear stress resulting from the viscosity increase of the organic phase, which facilitated the formation of emulsion droplets with larger size [25]. The polydispersity index (PDI) is a measure of dispersion homogeneity and usually ranges from 0 to 1. Values close to zero indicate a homogeneous dispersion, whereas those greater than 0.3 indicate high heterogeneity [29]. In this study, there were no significantly differences in PDI among nanoparticles made from different polymer types, and all the PDI values ranged from 0.2 to 0.3 indicating a narrow size distribution. As shown in Figure 3, all the Sal-LPNPs prepared by different polymers were negatively charged. It is well-known that nanoparticles with larger absolute value of zeta potential are more stable in colloidal dispersion system. Sal-LPNPs made by PLGA and PLGA-PEG-PLGA had higher absolute value of zeta potential, probably resulting from more remained PVA molecules in Sal-LPNPs. In this study, PVA [30] was used to contribute to the W/O/W double emulsion formation during Sal-LPNPs preparation. Different amount of PVA molecules would be inserted into the lipid bilayer due to the various interaction of PVA with copolymer, though most of the PVA molecules were removed by ultracentrifugation [31]. Part of chemical groups originated from the remained PVA molecules might be exposed on the surface of Sal-LPNPs. It is well-known that Zeta potential is the potential difference between the dispersion medium and the stationary layer of fluid attached to the dispersed nanoparticles [32]. Different surfaces of Sal-LPNPs would attach different amount of ions, thus Sal-LPNPs made from PLGA and PLGA-PEG-PLGA had higher absolute value of zeta potential.

**Figure 4.**
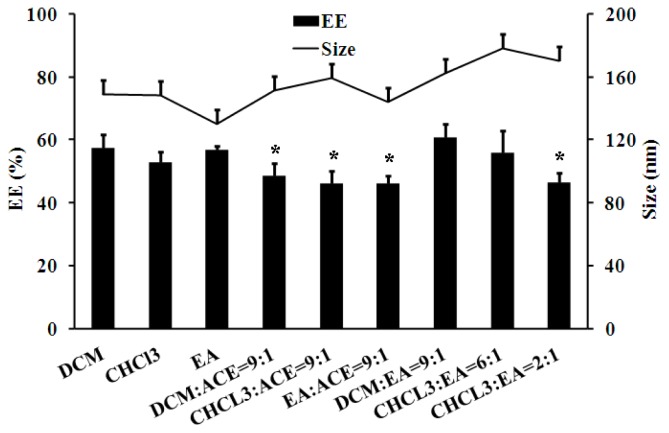
The effect of organic solvents on the entrapment efficiency (EE) and size of Sal-LPNPs (mean ± SD, *n* = 3). * indicated there was significant difference (*p* < 0.05) compared to DCM:EA = 9:1 group. DCM: dichloromethane; CHCl_3_: chloroform; EA: ethyl acetate; ACE: acetone.

The influence of the organic phase on Sal-LPNPs formulation was also investigated. There was almost no surface charge difference for Sal-LPNPs prepared by different organic phases (data not shown). The three organic phases containing single solvent had no significant effect on EE, but Sal-LPNPs with slightly smaller size can be obtained using ethyl acetate (EA). Sal-LPNPs were subsequently prepared using mixed organic solvents with different volume ratios. As shown in Figure 4, the introduction of acetone (ACE) dramatically decreased EE. ACE is a water-miscible organic solvent. Sal could escape from the inner aqueous phase into the outer aqueous phase mediated by ACE during the formation of W/O/W double emulsion [33,34]. Sal-LPNPs with highest EE and medium particle size were obtained when the mixture of dichloromethane (DCM)/EA (9:1, *v*/*v*) was used as the organic phase. The Sal diffusion from W/O/W double emulsion might be slowest under this condition.

**Figure 5.**
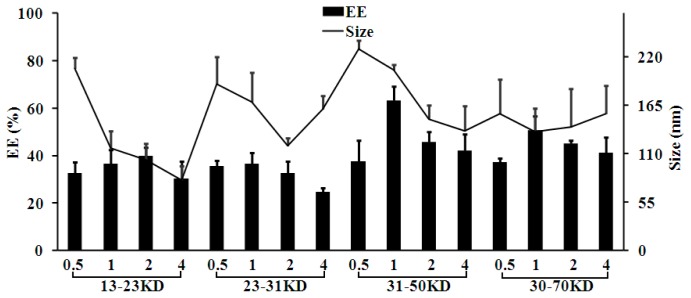
The effect of PVA on the entrapment efficiency (EE) and size of Sal-LPNPs (mean ± SD, *n* = 3). PVA molecular weight and concentration were shown on the *X* axis.

PVA is a widely used surfactant for constructing polymer nanoparticles. PVA types have significant effects on the pharmaceutical properties of nanoparticles. In this study, four kinds of PVA with different *M*_W_ were investigated at different concentrations in the outer aqueous phase. The zeta potentials of Sal-LPNPs kept consistent among different PVA types (data not shown), but the EE and particle size were significantly influenced. As shown in Figure 5, EE was slightly increased when the *M*_W_ of PVA increased*,* which might be caused by the raised viscosity of the aqueous phase. PVA concentrations had significant effects on particle diameters. The average size decreased with the increase of PVA content. As discussed in the literature [25], PVA can orient at O/W interface to facilitate the formation of smaller emulsion droplets at high concentration. EE changed slightly among different concentrations for low *M*_W_ PVA, whereas significant differences in EE were observed when high *M*_W_ PVA were used. PVA (1%, *w*/*v*) with *M*_W_ of 30–70 KD led to the highest EE, indicating that 1% PVA provided the best O/W interface in W/O/W double emulsion to prevent Sal diffusion from the W/O coarse emulsion.

According to the results described above, the optimal Sal-LPNPs were finally developed with sub-micron particle size as 155.25 ± 6.72 nm with polydispersity index (PDI) of 0.21 ± 0.15 and negative zeta potential of −22.8 ± 2.6 mV. The highest EE was 65.20% ± 1.34% (*n* = 3) and DL was 3.8% ± 0.03% (*n* = 3) using PLGA-PEG-PLGA (L:G = 3:1, 15 KD), mixed organic phase (DCM/EA = 9/1, *v*/*v*) and 1% PVA (*w*/*v*, 30–70 KD).

In our preliminary experiment, we prepared Sal-loaded PLGA nanoparticles and Sal-loaded liposomes using the same PLGA-PEG-PLGA and lipid components as in Sal-LPNPs. The maximal EE was only 28.03% ± 1.12% (*n* = 3) for Sal-loaded PLGA nanoparticles and 35.31% ± 0.98% (*n* = 3) for Sal loaded-liposomes, respectively. The data proved the LPNPs (the combination of polymer nanoparticles and liposome) indeed enhanced Sal EE. The combined utilization of polymer and lipids increased the diffusion pathway of Sal from W/O coarse emulsion and W/O/W double emulsion, thus synergistically improving Sal entrapment. Sal-LPNPs had similar EE to Sal-chitosan nanoparticles [17], but the anionic surface of Sal-LPNPs was obviously superior in antitumor therapy.

### 2.3. Transmission Electron Microscopy (TEM) Analysis

**Figure 6.**
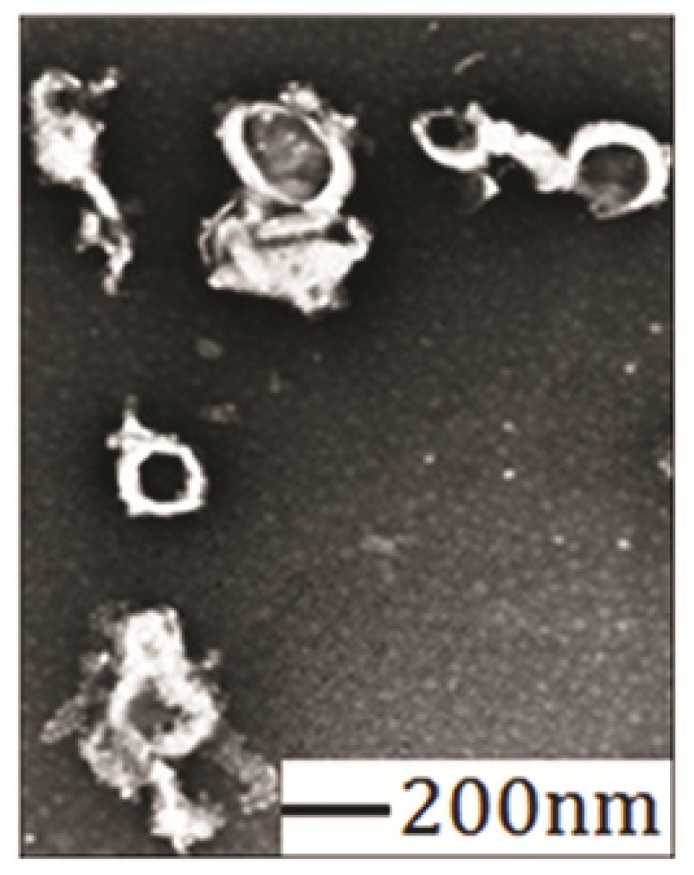
TEM image of Sal-LPNPs. Sal-LPNPs were general spherical with typical core-shell structure.

As shown in Figure 6, Sal-LPNPs were generally spherical. Obvious white shell structure was observed, indicating the core-shell structure of LPNPs. According to the literature [35–37], the dark core inside was composed of PLGA-PEG-PLGA copolymers and the white shell consisted of lipids. The mechanism of this core-shell formation might be rather complex [38], probably involving several interactions between lipid bilayers and the solid surfaces of the PLGA nanoparticles including Van der Waals, double layer, hydration, hydrophobic, thermal undulation and protrusion forces [39].

### 2.4. Differential Scanning Calorimetry (DSC) Analysis

**Figure 7.**
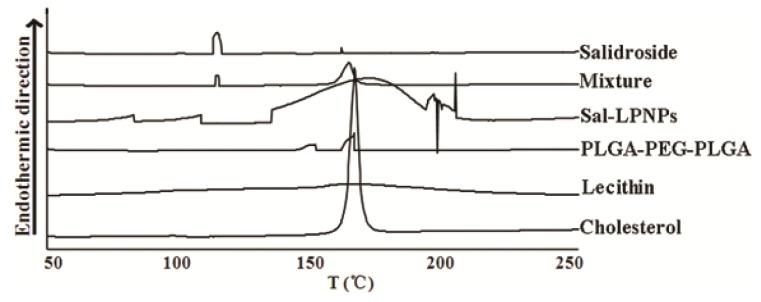
The DSC thermographs of Sal, Sal-LPNP, PLGA-PEG-PLGA, lecithin, cholesterol and the physical mixture of Sal, PLGA-PEG-PLGA, lecithin and cholesterol with the same mass ratios as those in Sal-LPNPs.

To characterize the existing state of Sal encapsulated in LPNPs, DSC analysis was performed [40]. As shown in Figure 7, the endothermic peaks of PLGA-PEG-PLGA, lecithin and cholesterol were at 148.07, 149.80 and 150.73 °C, respectively. Sal-LPNPs illustrated a smooth endothermic peak at 155.57 °C. The free Sal exhibited one obvious endothermic peak at 105.1 °C, which was also observed in the physical mixture but disappeared in Sal-LPNPs. The results indicated that Sal existed as an amorphous state or a solid solution in Sal-LPNPs and was successfully entrapped into the LPNPs.

### 2.5. *In Vitro* Drug Release

**Figure 8.**
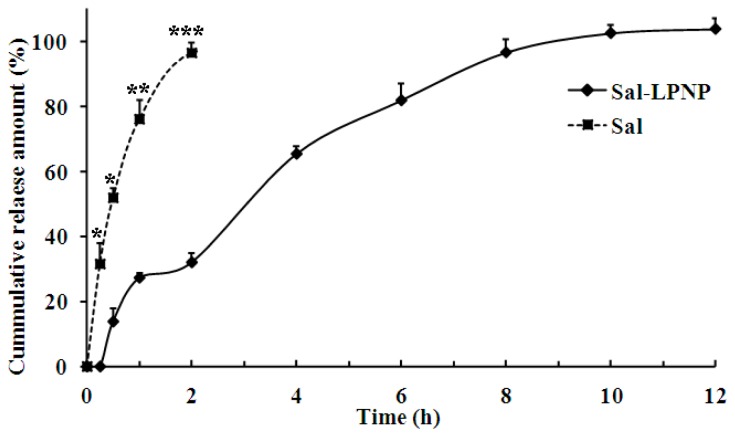
The *in vitro* release profiles of Sal and Sal-LPNPs in PBS (pH = 7.4, 0.01 M) at 37 °C. Free Sal released completely from the dialysis bag in 2 h while it took ~10 h to release the Sal from Sal-LPNPs (mean ± SD, *n* = 3). There were significant differences between Sal-LPNPs and free Sal groups at all time points before 2 h. * *p* < 0.05, ** *p* < 0.01, *** *p* < 0.001.

Free Sal (0.325 mg/mL), with the same concentration as Sal encapsulated in Sal-LPNPs, was placed into the dialysis bag to assess the retention effect of the dialysis bag itself. As shown in Figure 8, free Sal could be completely released from the dialysis bag in 2 h from pH 7.4 PBS, which demonstrated that this dialysis bag had a weak retention effect on Sal and was suitable to be used to assess the *in vitro* release profile of Sal out of Sal-LPNPs. Sal encapsulated in Sal-LPNPs only released 28% ± 1.21% (*n* = 3) in 2 h, which indicated that Sal-LPNPs were indeed sustained-release nanoparticles for Sal. Figure 8 displayed that Sal was gradually and slowly released from Sal-LPNPs. There was only 13.8% ± 1.89% (*n* = 3) Sal released from Sal-LPNPs in the first 0.5 h without apparent burst release, indicating most of Sal was entrapped in the core of Sal-LPNPs.

### 2.6. *In Vitro* Cell Viability

**Figure 9.**
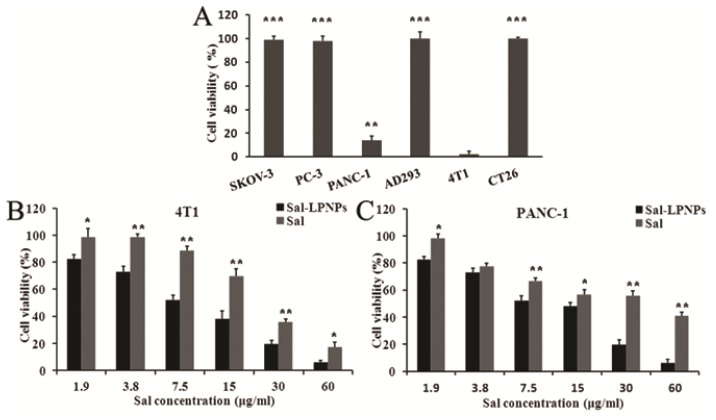
The cytotoxity of free Sal and Sal-LPNPs. (**A**) Three human originated cancer cell lines (SKOV-3, PC-3 and PANC-1), two mouse originated cancer cell lines (CT26 and 4T1) and one human normal cell line AD293 were treated with 100 μg/mL Sal for 48 h, respectively. * indicated there was significant difference (*p* < 0.05) compared to 4T1 cell line; (**B**) 4T1 cells were treated with Sal or Sal-LPNPs at different concentrations for 48 h; (**C**) PANC-1 cells were treated with Sal or Sal-LPNPs at different concentrations for 48 h. ** *p* < 0.01, *** *p* < 0.001.

Firstly, Sal sensitive cell lines were screened in six cells lines including three human originated cancer cell lines (SKOV-3, PC-3 and PANC-1), two mouse originated cancer cell lines (CT26 and 4T1) and one human normal cell line (AD293) by MTT method. Among them, the antitumor activity of Sal had only been reported in PANC-1 cells [41]. As shown in Figure 9A, no changes in growth were observed for SKOV-3, PC-3 and CT26 cells after treated with 100 μg/mL Sal for 48 h, indicating the insensitivity of these cancer cell lines to Sal. Meanwhile, the same concentration of Sal has no cytotoxicity to normal cell line AD293. However, the cell viabilities were significantly decreased for PANC-1 and 4T1 cell lines, indicating Sal might be a potential chemotherapeutic agent for pancreatic cancer and breast cancer. Therefore, the anticancer potential evaluation of Sal-LPNPs was further evaluated in PANC-1 and 4T1 cell lines.

The two cell lines were treated with free Sal, Sal-LPNPs or blank LPNPs. A range of 1.9–60 μg/mL Sal and corresponding amount of PLGA-PEG-PLGA copolymer and blank LPNPs were investigated. Blank LPNPs had no effect on cell growth for all the tested concentrations (data not shown). Free Sal and Sal-LPNPs inhibited the cell growth in a dose-dependent manner. Compared with free Sal groups, the cell viabilities were significantly decreased in Sal-LPNPs treated groups for both cell lines when Sal concentrations were above 3.8 μg/mL (Figure 9B,C). The *IC*_50_ values of free Sal and Sal-LPNPs in PANC-1 cell lines were 28.7 and 9.54 μg/mL, respectively, while those values in 4T1 cell lines were 24.6 and 8.23 μg/mL, respectively. There were almost three-fold differences in for both cell lines comparing free Sal and Sal-LPNPs, indicating the significantly increased antitumor activity of Sal-LPNPs. Nanoscale Sal-LPNPs with lipid shells could be efficiently taken up by cancer cells via endocytosis [42]. In contrast, the poor lipophilicity of free Sal led to low cancer cells uptake. Therefore, free Sal with higher concentration was needed to achieve the same antitumor efficacy as Sal-LPNPs.

## 3. Experimental Section

### 3.1. Materials

Salidroside (Sal, purity 98%) was purchased from National Institute for Drug and Food Control (Chengdu, China). 3-(4,5-dimethylthiazol-2-yl)-(2,5-diphenyl tetrazolium bromide) (MTT), PVA was purchased from Sigma (Shanghai, China). PLGA, PEG-PLGA, lactide and glycoside were acquired from the Changchun Shengbo Ma Biological Material Co. Ltd. (Changchun, China). PCL-PEG-PCL was kindly donated by Professor Zhiyong Qian (Sichuan University, Sichuan, China). Lecithin was purchased from Shanghai TaiWei Co. Ltd. (Shanghai, China). Cholesterol was obtained from Shanghai Bio Life Science & Technology Co. Ltd. (Shanghai, China). All the other chemicals were of analytical grade.

### 3.2. Cell Lines and Culture

All the cell lines were obtained from ATCC (Manassas, VA, USA). The mouse colon cancer CT26 cell line and mouse breast cancer 4T1 cell line were cultivated in RPMI-1640 (Gibco-BRL, Grand Island, NE, USA). The human ovarian cancer SKOV-3 cell line, human pancreatic cancer PANC-1 cell line, human prostate cancer PC-3 cell line and human normal AD293 cell line were incubated in DMEM (Gibco-BRL, Grand Island, NE, USA) medium. Both mediums were supplemented with 10% fetal bovine serum and 0.1% penicillin G sodium. All the cells were cultured at 37 °C in a humidified incubator with 5% CO_2_.

### 3.3. Synthesis of PLGA-PEG-PLGA Triblock Copolymer

PLGA-PEG-PLGA triblock copolymers were synthesized by a typical ring open copolymerization of lactide and glycoside using hydroxyl-terminated PEG as initiator [23,43,44]. Briefly, 3 g PEG was dried by continuous stirring under vacuum at 150 °C for 3 h. LA (5.7 g) and GA (1.75 g) were then added in nitrogen atmosphere and stirred under vacuum at 120 °C. After all the monomers were melted, stannous octoate (0.2% *w*/*v*) was added and the reaction mixture was stirred under vacuum at high temperature for several hours. Then the products were dispersed in cold water (5–8 °C). The obtained suspension was heated to 80 °C to precipitate the polymer product and remove low molecular weight water-soluble polymers and unreacted monomers. The above purification process was repeated twice to obtain PLGA-PEG-PLGA with high purity. Finally, the residual water was removed by freeze-drying. PLGA-PEG-PLGA was confirmed by ^1^H NMR. ^1^H NMR spectra were recorded at room temperature on a BrukerAvance III spectrometer (400 MHz for ^1^H NMR spectroscopy, Bruker Scientific Technology Co., Ltd., Beijing, China), using tetramethylsilane (TMS) as an internal standard and CDCl_3_ as solvent.

### 3.4. Preparation of Sal-LPNPs

**Figure 10.**
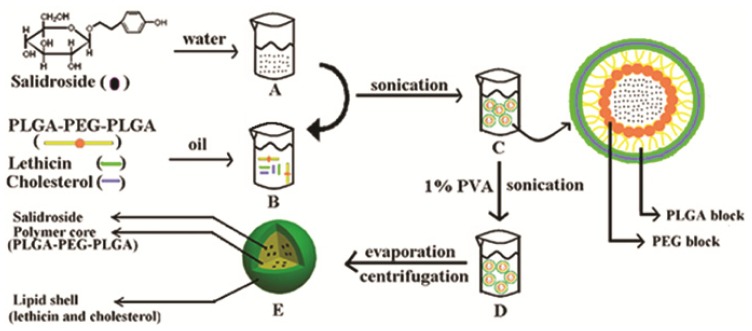
The schematic diagram of Sal-LPNPs preparation. Firstly, Sal was dissolved in water to form an internal aqueous phase (**A**); The organic phase was composed of PLGA-PEG-PLGA, cholesterol and lecithin in dichloromethane (**B**); Secondly, the internal aqueous phase was added into the organic phase to get the coarse emulsion by sonication (**C**); Thirdly, 1% PVA (*M*_W_ = 30–70 KD) solution was added into the coarse emulsion to further form W/O/W double emulsion by sonication (**D**); Then the organic solvent was removed by rotary vacuum evaporation. Lastly, free Sal was removed by centrifugation to get the Sal-LPNPs (**E**).

Sal-LPNPs were prepared by the W/O/W double emulsification method. In this study, the effect of the polymer types, organic phase, PVA types and amount on nanoparticles mean diameter and drug entrapment efficiencies were assessed. Unless otherwise mentioned, all the experiments were conducted by varying one of the parameters while keeping all the other process parameters at a set of standard conditions: Firstly, 40 mg PLGA-PEG-PLGA copolymer and 10 mg lipids mixture (lecithin/cholesterol = 5/1, *w*/*w*) [24] were dissolved in 1 mL dichloromethane as the organic phase. Then the organic phase was emulsified with the internal aqueous phase containing 0.2 mL Sal solution (10 mg/mL) by microtip probe sonicator in ice bath at 55 W for 1 min with 5 s pulse-on and 5 s pulse-off. After the formation of W/O coarse emulsion, 4 mL of 1% PVA (*M*_W_ = 30–70 KD) solution was added into the coarse emulsion to further get W/O/W double emulsion by sonication (65 W for 1 min with 5 s pulse-on and 5 s pulse-off). Then the organic solvent was immediately removed by rotary vacuum evaporation. Then the solution was centrifuged for 70 min (1.20 × 10^4^ g/min) at 4 °C to remove the PVA and free Sal. The precipitate was dissolved in PBS (pH 7.4, 0.01 M). The obtained Sal-LPNPs were stored at 4 °C for further evaluation. The schematic diagram of optimal Sal-LPNPs preparation was shown in Figure 10.

### 3.5. Particle Size and Zeta Potential

The particle size, distribution and zeta potential were determined by dynamic light scattering (DLS) with a Zetasizer Nano Zen-3600 instrument (Malvern Instruments, Malvern, UK). The Sal-LPNPs were 10 times diluted with de-ionized water for measurement at 25 °C.

### 3.6. Encapsulation Efficiency (EE) and Drug Loading (DL)

To determine EE, 1 mL Sal-LPNPs solution was centrifuged for 70 min (1.20 × 10^4^ g/min) at 4 °C. The precipitate was dissolved in DMSO. The amount of Sal in precipitate was defined as the capsulated part and determined by high-performance liquid chromatography (HPLC) assay (Waters e2695, Waters Co., Shanghai, China). A reverse-phase Cosmosil C18 column (150 × 4.6 mm, pore size 5 μm, Global chromatography) was used for the chromatographic separation with an injection volume of 20 μL. The mobile phase was a mixture of methanol/water (75/25, *v*/*v*) at a rate of 1.0 mL/min. Sal was detected at 278 nm [45].

(1) $$\text{Encapsulation efficiency }(\text{EE})\;(\%)=\frac{\text{amount of the Sal in Sal}-\text{LPNPs}}{\text{initial amount}}\times 100\%$$

(2) $$\text{Drug loading }(\text{DL})\;(\%)=\frac{\text{weight of the drug in Sal}-\text{LPNPs}}{\text{weight of the feeding polymer and drug}}\times 100\%$$

### 3.7. Transmission Electron Microscopy (TEM) Analysis

Before analysis, the Sal-LPNPs were diluted with purified water and negatively stained with 2% (*w*/*v*) phosphotungstic acid for 30 s. Then they were placed on copper grids precoated with a thin film of poly-vinylformaldehyde and operated at 120 kV for observation (H-600, Hitachi, Tokyo, Japan).

### 3.8. Differential Scanning Calorimetry (DSC) Analysis

The physical state of Sal-LPNPs was investigated by DSC (DSC 200PC, Netzsch, Karlsruhe, Germany) under nitrogen atmosphere at a flow rate of 20 mL/min. Freeze-dried Sal-LPNPs without cryoprotectant, free Sal, PLGA-PEG-PLGA, lecithin, cholesterol and the physical mixture of the latter four with the same mass ratios as those in Sal-LPNPs were heated from 50 to 250 °C and the scanning rate was set at 10 °C/min.

### 3.9. *In Vitro* Drug Release

The release profile of Sal-LPNPs was investigated in PBS buffer by dialysis method. Briefly, 1 mL Sal-LPNPs or 1 mL of 0.325 mg/mL free Sal solution (Sal was directly dissolved in pH 7.4 PBS) was placed in a dialysis bag (molecular weight cut off: 3 KD; Green Bird, Shanghai, China), which was put into 35 mL PBS at 37 °C with shaking rate of 100 rpm. At given time points, 0.2 mL buffer was taken from the medium. Same amount of fresh medium was added while samples were taken out. The content of Sal was measured by HPLC.

### 3.10. *In Vitro* Cell Viability

Cell viability of blank-LPNPs, Sal and Sal-LPNPs were evaluated by MTT assay [46] using six cell lines including three human originated cancer cell lines (SKOV-3, PC-3 and PANC-1), two mouse originated cancer cell lines (CT26 and 4T1) and one human normal cell line (AD293). The cells (100 μL medium, 4 × 10^4^ cells/mL) were plated in 96-well plates and incubated for 24 h at 37 °C in humanized atmosphere containing 5% CO_2_. Then they were treated with blank-LPNPs, Sal or Sal-LPNPs at different concentrations for 48 h under the same condition. After incubation, 20 μL MTT (5 mg/mL, dissolved in saline) was added to each well and incubated for another 4 h. The incubation medium was removed. DMSO (150 μL) was added to each well and gently shaken for 10 min at 25 °C. The absorbance was measured at 570 nm (Multiskan MK3, Thermo Scientific, Waltham, MA, USA). The absorbance value of untreated cells was set as 100% for reference. Each experiment was repeated three times in three wells. The *IC*_50_ values of blank-LPNPs, Sal or Sal-LPNPs were calculated from cytotoxicity data by *IC*_50_ computational software (SPSS V 17.0, IBM Corp., New York, NY, USA).

### 3.11. Statistical Analysis

The mean values were obtained as an average of three different preparations. Comparisons were performed using independent *t*-test using SPSS (V 17.0, IBM Corp., New York, NY, USA). Data were expressed as means ± standard deviation. A *p* value of less than 0.05 is considered as statistically significant.

## 4. Conclusions

Sal, a water-soluble drug, was successfully loaded into LPNPs consisting of PLGA-PEG-PLGA and lipids (lecithin and cholesterol) with high EE (65%) and negative surface charge. The core-shell structure of Sal-LPNPs was verified by TEM. Sal could be gradually released from LPNPs without burst release. Sal-LPNPs significantly improved the *in vitro* antitumor activity of Sal in PANC-1 and 4T1 cancer cell lines. Sal-LPNPs could selectively kill tumor cells but not normal cells *in vivo* because Sal had no toxicity on AD293 cells at a concentration (100 μg/mL). Moreover, Sal-LPNPs might achieve higher antitumor efficacy than free Sal by accumulating specifically in tumor tissues due to EPR effect. Thus, LPNPs are a promising Sal vehicle for anti-cancer therapy and worthy of further investigation.
